# Enhancing health outcomes through genetic-based personalized nutrition: investigating the effects of dietary behavior change

**DOI:** 10.1186/s40795-026-01311-6

**Published:** 2026-04-07

**Authors:** Hector Guillen-Ahlers, Rajni Nigam, Hannah Pekarek, Kelly Van Gorden, Frankie O’Brien, Kristin Ricklefs-Johnson, Jignesh M. Patel, Yi Zhang

**Affiliations:** 1 GenoPalate Inc., 10437 W Innovation Drive, Suite #465, Milwaukee, WI 53226 USA; 2 National Dairy Council, Dairy Management Inc., Rosemont, IL USA; 3 Carnegie Mellon University, Pittsburgh, PA USA; 4 DataChat Inc. NDC, Madison, WI USA; 5 Buck Institute for Research on Aging, Novato, CA USA

**Keywords:** Direct-to-consumer genetic testing, Behavioral changes, Personalized nutrition, Metabolic health, Nutrigenomics

## Abstract

**Background:**

Personalized nutrition, an approach using individual-specific information to tailor dietary recommendations, has emerged as a promising strategy for promoting healthier eating behaviors and improving health outcomes. This study aimed to assess the impact of genetically informed dietary behavior changes on health improvement.

**Methods:**

Data related to behavior changes and health improvements were collected from 874 research participants who had received a genetics-based personalized nutrition report for at least one year, as part of a retrospective cohort study. Changes related to improvements in health associated with behavioral change and key genotypes were analyzed using chi-square tests, paired t-tests, one-way ANOVA, and linear and logistic regression models.

**Results:**

Behavior changes had a significant impact on participants’ health, with an average of 57% reporting health improvement, compared to 12% for those who did not modify their behaviors (*p* < 0.001). Weight changes, albeit modest, were also significantly different, with individuals averaging a weight change of –0.5% and + 1.5% for those who changed and did not change their behavior, respectively. The results showed segregation according to key genotypes in the *FTO, TCFL2, LEPR,* and *PPARG* genes. The duration of the behavior change also showed a positive effect on health improvement rates.

**Conclusion:**

Findings suggest that an actionable, genetics-based personalized nutrition service can help foster sustainable behavioral changes, leading to higher rates of health improvements.

**Supplementary Information:**

The online version contains supplementary material available at 10.1186/s40795-026-01311-6.

## Background

The value of nutrigenomics as part of personalized nutrition approaches aligns with the potential recognized in personalized medicine. It is garnering attention, not only to improve dietary guidance but also as a mechanism to motivate desirable behavioral changes [1]. Studies have shown that changes to dietary and lifestyle behaviors occur among users of direct-to-consumer genetic testing services (DTC-GTS), albeit in modest proportions [1–3]. Research on the impact of personalized nutrition on behavior and health is still limited. However, it is becoming evident that one-size-fits-all dietary recommendations are often inadequate [4], and a higher level of personalization can lead to more sustainable changes [5]. Naturally occurring genetic variations in the form of single nucleotide polymorphisms (SNPs) influence an individual’s response to specific nutrients and dietary patterns [6–12]. Incorporating genetics into personalized nutrition has the potential to improve health outcomes by positively altering behavior [13].

SNPs of interest can be genotyped to tailor dietary recommendations based on these genetic variants [7]. Carriers of the minor allele at rs9939609 in the *FTO* gene show a decreased risk for obesity when following low-fat (13.4–35.5% of energy) or high-carb (48.3–73.2% of energy) diets [6]. Another *FTO* polymorphism (rs8050136) also shows a decrease in obesity with high fiber intake (< 14 g) among minor allele carriers [8]. Similarly, the major allele at rs1137101 in the *LEPR* gene also shows an increased risk of obesity for elevated intakes of total fat (≥ 83 g/d) and saturated fat (≥ 12 g/d) [9]. Delivering these kinds of evidence-based associations between genotypes and metabolic responses to dietary factors has the potential to empower DTC-GTS users to weigh individual genetic insights to prioritize changes more likely to be impactful to them. It has been shown that users can effectively comprehend genetic results [14], and the notion that DTC-GTS generates anxiety among its users has largely been disproved [15, 16]. There is an appetite in the population for these kinds of services, reflected in the growing global landscape of nutrition-specific DTC-GTS [17].

The present study aimed to assess whether behavior changes among users of a DTC-GTS solution for personalized nutrition through self-report surveys translate into higher rates of health improvements. It has been highlighted that research focused on using mobile and wireless technologies to facilitate dietary interventions has been insufficient, with short durations and low participation numbers [18], both of which are addressed herein. To the best of our knowledge, this is the first study looking at the effect of nutrition-centric DTC-GTS-driven behavior change on general health improvement among its users.

## Methods

### Study participants

The Genetic-Enabled Nutritional Output toward Food as Medicine (GENOFAM) study aims to understand the impact of genetic-based personalized nutrition information on its users. Enrolled research participants (69,570) come from GenoPalate, a data-driven health company that offers services on personalized nutrition, defined as using individual-specific information, founded in evidence-based science, to promote dietary behavior change that may result in measurable health benefits [19]. All users come from the USA, and except for Hawaii, all states were represented among survey respondents (Figure S1). An Ethical and Independent Review Board approved all procedures involving research study participants. Consent to participate in research was captured electronically. During our analysis, de-identified, encrypted IDs were used to maintain confidentiality and protect personal data. Guidelines laid down in the Declaration of Helsinki were followed. Cohort characteristics are displayed in Table 1. Only users who had received their nutritional report for at least one year and had completed the onboarding survey (47,299) were recruited via email to participate in a follow-up survey. The single recruitment touchpoint limited the survey participation rate (1.8%). Those who did not opt in to participate in research were removed from all analyses included in this study. To reach the target participation rate based on the power parameters described below, $5 gift cards were offered as incentives in a re-targeting effort. A direct comparison between those with (29%) and without (71%) incentives is presented in Table S1. No statistically significant differences were observed between the two groups, except for age. The incentive group was significantly younger (44.9 ± 12.0 years) than the no-incentive group (54.1 ± 12.4 years), with a mean difference of 9.25 years (95% CI [7.41, 10.99]; t(462) = 10.10, *p* < 0.001).

**Table 1:** Cohort characteristics

	**Follow up survey respondents (*n* = 874)**
Female, n (%)	631 (73%)
BMI, mean ± sd	29.3 ± 6.5
Age, mean ± sd	51.5 ± 13
Age group, n (%)	
18–24	7 (1%)
25–34	87 (10%)
35–44	181 (21%)
45–54	225 (26%)
55–64	208 (24%)
65 +	161 (19%)
Self-reported ethnicity, n (%)	
White, non-Hispanic	744 (77.2%)
Hispanic or Latino	85 (8.8%)
Black	39 (4.0%)
Asian	30 (3.1%)
Native American/Alaskan Native	26 (2.7%)
Other	20 (2.1%)
Middle Eastern	16 (1.7%)
Indian	4 (0.4%)

BMI measured in kg/m^2^. sd denotes standard deviation

### Personalized nutritional recommendations

GenoPalate gathers information about its customers, including sex, age, dietary preferences (vegan, pescatarian, etc.), average exercise habits, and habits related to diet (average intake of fruits and vegetables, eating out frequency, foods avoided in their diet, etc.). Once DNA genotypes are obtained, their personalized nutritional recommendations are computed by GenoPalate's nutritional algorithm and delivered to customers. The online report provides detailed genetic insights, nutritional recommendations, and a list of scored foods based on each user's genetic profile and collected data (Supplementary file). The nutritional report is based on peer-reviewed research studies and include recommendations for carbohydrates (total carbohydrates, fiber and sugar), fats (total fats, mono- and polyunsaturated fatty acids, saturated fats), protein, vitamins B6, B12, A, D, E and C, folate, magnesium, iron, sodium, calcium, potassium, selenium and zinc. A total of 153 SNPs are used in the full report, which also provides insights into eating behaviors. The nutritional report relies on 97 SNPs related to 44 genes to produce these traits mentioned above. In the absence of impactful variants for a specific trait, the nutritional recommendations are based on guidelines from the National Institutes of Health, which account for age and sex. Broadly driven by minor allele frequencies, on average, 40% of a user’s traits will not have an impactful variant. Depending on the nutrient, recommendations are based on the dietary guidelines for Americans [20], the recommended daily allowance, the adequate intake level, or acceptable macronutrient distribution range [21]. A detailed description of the recommended foods and their beneficial nutrients is presented to the user to help them understand why the recommendations are made. In addition, other genetic insights related to eating behaviors and possible food sensitivities (such as lactose and gluten) are also presented to the user.

### Data collection

Data related to nutritional intake, environmental factors, eating behavior, and social determinants of health were collected electronically through onboarding surveys consisting of qualitative and quantitative measures. During this process, users were asked if they would like to participate in research and were given the option to change their choice at any point directly from their accounts. The follow-up assessment consisted of a short survey that participants completed electronically, which included options for self-reported behavior changes, health improvements, and current weight. With few exceptions, the survey consisted of multiple-choice answers. The onboarding and follow-up questions used in this study are presented in Figure S2. The survey also investigated other topics (outside the scope of this paper) related to reasons for the lack of behavior change, helpful parts of the report, and desired changes within the service. In the follow-up survey, two response options to the behavior change question, (1) increased fruit and/or vegetable consumption and (2) decreased snacking on foods high in sodium, saturated fat, and added sugars, were used as proxies to qualitatively assess directional changes in fiber intake, the balance of carbohydrate and fat intake, and intake of saturated fat and sugar, respectively.

### Statistical analysis

All statistical analyses and data visualizations were generated using R version 4.2.1. Data pre-processing was performed using DataChat Inc. Power calculations were conducted with the aid of G*Power 3.1 [23], targeting a sensitivity of 0.15, assessed through a chi-square analysis with an alpha of 0.05, a beta of 0.8, and up to 14 degrees of freedom. For analyses involving weight changes, samples beyond four standard deviations (~ 34% change) in either direction were removed, as weights could not be corroborated, and there were more cases than a normal distribution would predict, thereby increasing the risk of inaccuracy in those cases. Participants with missing values in health improvements (n = 12), behavior change (n = 5), and weight (n = 76) questions were retained and excluded only from analyses that required those answers. Pearson's chi-square tests were used to assess the statistical significance of categorical data. Two-sided Student’s t-tests and 1-way ANOVA were used to determine the significance between continuous variables. Linear and logistic regressions were used to assess covariates. A three-factor ANOVA was used to determine the effect of several independent variables on BMI. In all analyses, a p-value below 0.05 was considered significant. All confidence intervals (CI) were calculated for an alpha of 0.05.

## Results

### Behavior changes lead to health improvements

**Fig. 1:**
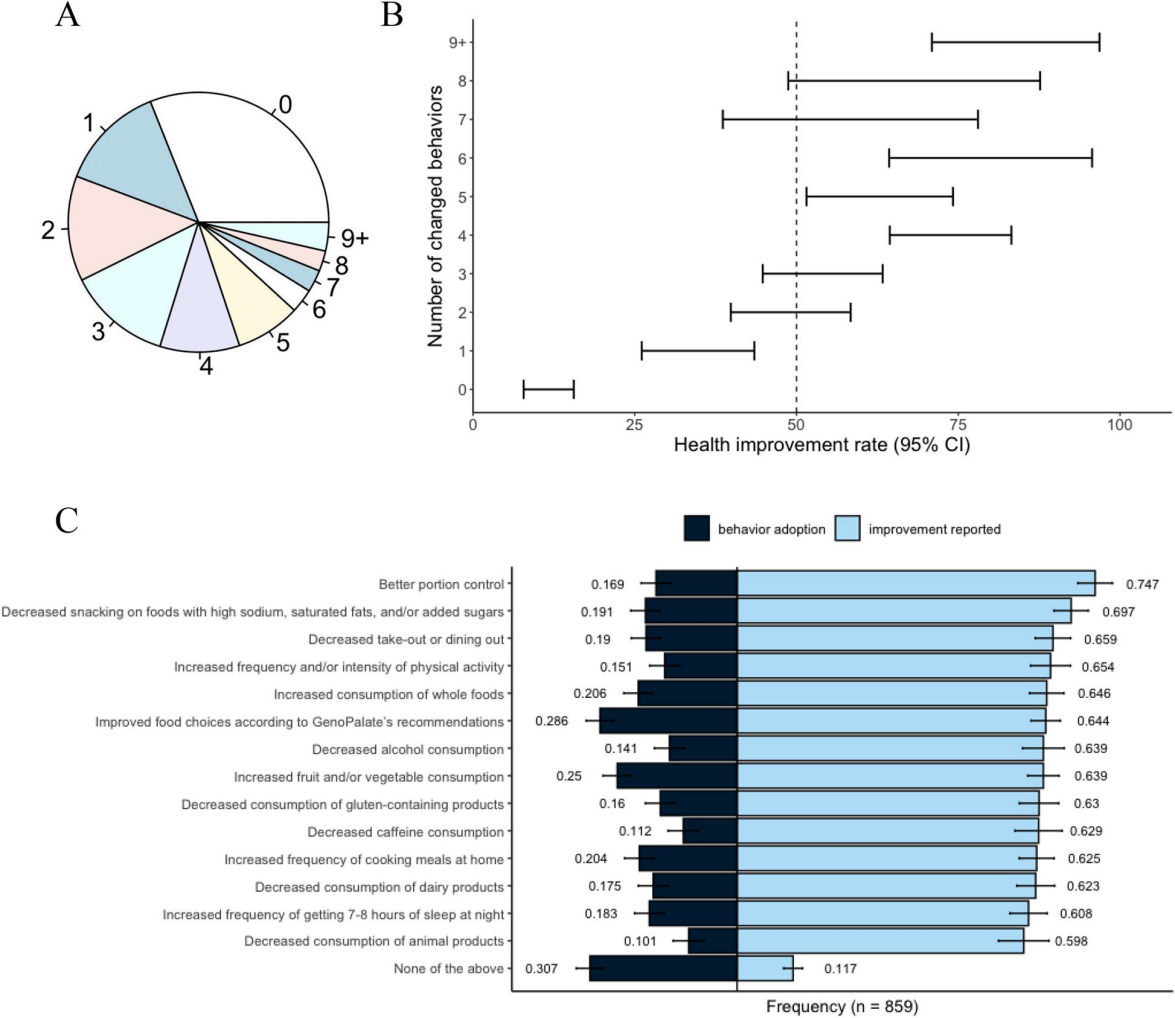
Behavior changes and health improvement rates. **A** Distribution of participants according to the number of behavior changes reported (*n* = 870). **B** Improvement rate confidence intervals at an alpha of 5% according to the number of behavior changes selected (*n* = 859). **C** Selection frequency of each behavior change option (dark blue bars) and reported health improvement proportion shown for each of those behavior change options (light blue) (*n* = 859). Error bars represent standard error

Eligibility was limited to users who had received their report at least one year prior to the study (mean = 2.05 ± 0.5 years). Participants were allowed to select multiple behavior changes (Fig. 1A, Figure S2), and results were correlated with health improvements (Fig. 1B-C and Table S2). Behavior changes were qualitative, and users who did not select one of the behavior change options (‘None of the above’) were categorized as non-behavior changers. BMI, age, and sex did not significantly differ among behavior and non-behavior changers (Table 2). Certain behaviors that the users had reported during onboarding (fruit and vegetable intake, frequency and intensity of physical activity, and frequency of eating out) also did not show significant differences (Table 2 and Table S3). Rates of self-reported health improvements (listed in Table 3) were significantly higher in the behavior change group (57 ± 0.04% vs 12 ± 0.039%, n = 859, χ2_1_ = 148.9, *p* < 0.001). Those who changed behavior lost an average of 0.8 kg (± 7.9), compared to 1.0 kg (± 5.3) gained by those stating not having changed behavior (t(674) = 3.54, p < 0.001), with a mean difference of 1.95 kg (95% CI [0.87, 3.03]). This observation coincided with weight loss/management, singled out as the most frequently reported health improvement (21%), and was not affected by starting BMI values, which were very similar between both groups (t(596) = 0.89, p = 0.37, mean difference = 0.42 kg/m2, 95% CI [–1.34, 0.50]) (Table 2). Users who did not experience health improvements were allowed to differentiate if they did not have a need for any of the options given (Figure S2). The latter was significantly higher (19 ± 0.047% vs. 10 ± 0.024%, χ2_1_ = 13.22, *p* < 0.001) among those who reported not having changed their behavior.

**Table 2:** Cohort characteristics stratified by behavior change adoption

	**Entire cohort**	**No behavior change**	**Behavior change**	**p**
n	874 (100%)	270 (31%)	600 (69%)	
Female, n (%)	636 (73%)	198 (74%)	436 (73%)	0.837*
Health improvement, p ± 95% CI	0.43 ± 0.033	0.12 ± 0.039	0.57 ± 0.04	< 0.001*
No need for health improvement, p ± 95% CI	0.12 ± 0.022	0.19 ± 0.047	0.1 ± 0.024	< 0.001*
BMI, mean ± sd	29.5 ± 6.8	29.2 ± 6.1	29.6 ± 7.1	0.373‡
Age, mean ± sd	51.6 ± 13.3	51.4 ± 12.5	51.7 ± 13.7	0.807‡
Weight change (kg), mean ± sd	−0.2 ± 7.6	1.3 ± 5.6	−0.9 ± 8.3	** < 0.001‡**
Weight change (%), mean ± sd	0.1 ± 8.4	1.5 ± 6.6	−0.5 ± 9	**0.004‡**
Initial fruit/vegetable intake				0.049*
0–1 servings	189 (21.6%)	63 (23.3%)	126 (21.0%)	
2–3 servings	446 (51.0%)	121 (44.8%)	322 (53.7%)	
4–5 servings	191 (21.9%)	65 (24.1%)	125 (20.8%)	
6 or more servings	48 (5.5%)	21 (7.8%)	27 (4.5%)	
Self-reported ethnicity				0.26*
White, non-Hispanic	746 (77.3%)	229 (76.8%)	517 (77.5%)	
Hispanic or Latino	84 (8.7%)	25 (8.4%)	59 (8.8%)	
Black	39 (4.0%)	15 (5.0%)	24 (3.6%)	
Asian	30 (3.1%)	5 (1.7%)	25 (3.7%)	
Native American/Alaskan Native	26 (2.7%)	7 (2.3%)	19 (2.8%)	
Other	20 (2.1%)	8 (2.7%)	12 (1.8%)	
Middle Eastern	16 (1.7%)	6 (2.0%)	10 (1.5%)	
Indian	4 (0.4%)	3 (1.0%)	1 (0.1%)	

BMI measured in kg/m^2^. Significant differences highlighted in bold. P denotes proportion. CI denotes confidence intervals with an alpha of 0.05. sd denotes standard deviation

^*^Chi-square used to obtain p-values between ‘No behavior change’ and ‘Behavior change’ groups

^‡^Student t used to obtain p-values between ‘No behavior change’ and ‘Behavior change’ groups

**Table 3:** Distribution of health improvement options selected from the follow-up survey

Response Options	N (%)
Weight loss or better weight management	181 (20.97%)
Better digestion	122 (14.14%)
Better sleep	110 (12.75%)
Decreased pain or inflammation	93 (10.78%)
Better mood	73 (8.46%)
Decreased or improved management of blood sugar	69 (8%)
Decreased stress levels	63 (7.3%)
Decreased or improved management of cholesterol	53 (6.14%)
Other	43 (4.98%)
Decreased or improved management of blood pressure	40 (4.63%)
Nothing (no progress since joining GenoPalate)	399 (46.23%)
Nothing (did not have a need for any of them)	107 (12.4%)

Multiple selections were allowed

Of the users who reported a behavior change, 36% reported that GenoPalate had helped, and 43% reported that it had partially helped them achieve this change. (Table 4). Self-reported health improvement was significantly different among the three groups (No, Partially, Yes), with the highest rate (0.73 ± 0.06) reported among those who found GenoPalate helpful and the lowest (0.37 ± 0.09) among those who did not (χ2_2_ = 48.2, *p* < 0.001). BMI, sex, weight change, and relevant starting behaviors did not show significant differences between the three groups. A one-way ANOVA revealed a significant age difference, with a tendency for younger age averages (49.3 ± 13.0) among those who found the genetic service helpful in making their behavioral changes (F(2, 589) = 4.82, *p* = 0.008) (Table 4).

**Table 4:** Characteristics of users according to how helpful they considered the personalized nutrition service

	**No**	**Partially**	**Yes**	**p**
n (%)	125 (21%)	255 (43%)	212 (36%)	
Female, n (%)	100 (80%)	180 (71%)	149 (70%)	0.105*
Health improvement, p ± 95% CI	0.37 ± 0.085	0.52 ± 0.061	0.73 ± 0.06	< 0.001*
No need for health improvement, p ± 95% CI	0.09 ± 0.05	0.11 ± 0.038	0.08 ± 0.037	0.531*
BMI, mean ± sd	29.7 ± 8	29.6 ± 6.2	29.4 ± 7.6	0.922^§^
Weight change (kg), mean ± sd	−0.2 ± 10.4	−0.6 ± 6.8	−1.4 ± 7.3	0.416^§^
Weight change (%), mean ± sd	0.1 ± 10.7	−0.3 ± 7.5	−0.9 ± 8.2	0.557^§^
Age, mean ± sd	53.7 ± 11.9	52.4 ± 14.8	49.3 ± 13	**0.008**^**§**^

BMI measured in kg/m^2^. Significant differences highlighted in bold. p denotes proportion. CI denotes confidence intervals with an alpha of 0.05. sd denotes standard deviation

^*^ Chi-square used to obtain p-values

^§^ 1-way ANOVA used to obtain p-values

### Genotype impact of behavior changes on weight

Habit changes related to dietary fiber, saturated fat, and carbohydrate intake were inferred based on fruit and vegetable consumption and unhealthy snacking. Weight changes were segregated based on relevant genotypes in these traits previously shown to affect weight, as a function of these dietary habits [6, 8–10, 12, 22, 23] (Table 5). G allele carriers for rs1137101 in the *LEPR* gene showed statistically significant (t(250.6) = −2.19, p = 0.03, 95% CI [−2.83, −0.15]) weight loss (−1.38 ± 0.59 kg) after an increase in fruit and vegetable intake compared to those who did not report that change (+ 0.11 ± 0.35 kg). No significant weight change was observed in the AA group (t(100.9) = −0.13, p = 0.896, 95% CI [−2.5, 2.19]). Similar trends were observed among A allele carriers at rs8050136 (−1.57 ± 0.63 kg, t(244) = −2.09, p = 0.038, 95% CI [−3.02, −0.09]) and rs9939609 (−1.61 ± 0.62 kg, t(243.3) = −2.22, p = 0.027, 95% CI [−3.1, −0.19]) in the *FTO* gene. Similarly, a decrease in unhealthy snacking (high in added sugars, salt, and saturated fat), displayed statistically significant weight loss in risk allele carriers of the same three SNPs (rs1137101, −1.98 ± 0.72 kg, t(150.5) = −2.66, p = 0.009, 95% CI [−3.67, −0.54]; rs8050136, −2.39 ± 0.71 kg, t(176.7) = −3.11, p = 0.002, 95% CI [−4.13, −0.92]; rs9939609, −2.43 ± 0.71 kg, t(177) = −3.25, p = 0.001, 95% CI [−4.19, −1.02]), while variants in rs1801282, rs7903146, and rs5082 in the *PPARG, TCFL2* and *APOA2* genes, respectively, failed to show differences attributable to their genotypes. All interaction p-values exceeded 0.05 and were therefore not reported. Given the directional and qualitative nature of the behavioral outcomes, interaction terms were not expected to yield meaningful interpretive value in this study.

**Table 5:** Weight loss related to behavior change and genotype

**Polymorphism**	**Risk** **allele**	**Increased intake of fruits and vegetables**			**Decreased unhealth snacking**		
		**No**	**Yes**		**No**	**Yes**	
		**△ Kg ± SE**		**p**	**△ Kg ± SE**		**p**
rs9939609							
AA + AT (*n* = 507)	A	0.03 ± 0.4	−1.61 ± 0.62	**0.027**	0.18 ± 0.38	−2.43 ± 0.71	**0.001**
TT (*n* = 271)		0 ± 0.45	−0.12 ± 0.88	0.904	0.11 ± 0.44	−0.74 ± 0.99	0.431
rs7903146							
CC + CT (*n* = 723)	C	−0.01 ± 0.32	−0.97 ± 0.54	0.126	0.11 ± 0.3	−1.79 ± 0.62	**0.006**
TT (*n* = 55)		0.5 ± 0.93	−1.98 ± 1.57	0.183	0.93 ± 0.83	−2.99 ± 1.71	**0.048**
rs1801282							
GG + GC (*n* = 166)	G	1.03 ± 0.63	−0.55 ± 0.82	0.13	1.21 ± 0.54	−2.1 ± 1.35	**0.028**
CC (*n* = 612)		−0.25 ± 0.34	−1.23 ± 0.61	0.165	−0.14 ± 0.34	−1.9 ± 0.64	**0.016**
rs1137101							
GG + GA (*n* = 550)	G	0.11 ± 0.35	−1.38 ± 0.59	**0.03**	0.13 ± 0.33	−1.98 ± 0.72	**0.009**
AA (n = 228)		−0.2 ± 0.6	−0.35 ± 1.02	0.896	0.22 ± 0.6	−1.86 ± 0.98	0.074
rs8050136							
AA + AC (n = 502)	A	−0.02 ± 0.41	−1.57 ± 0.63	**0.038**	0.13 ± 0.39	−2.39 ± 0.71	**0.002**
CC (n = 276)		0.09 ± 0.43	−0.21 ± 0.87	0.758	0.19 ± 0.43	−0.88 ± 0.97	0.32
rs5082							
GG + GA (n = 461)	G	0.26 ± 0.37	−0.96 ± 0.66	0.112	0.3 ± 0.36	−1.67 ± 0.79	**0.024**
AA (n = 317)		−0.32 ± 0.51	−1.27 ± 0.81	0.319	−0.08 ± 0.49	−2.28 ± 0.86	**0.028**

**△** Kg denotes the difference in weight (in kilograms). SE denotes standard error. Student's t-test is used to obtain p-values. All interaction *p*-values > 0.05 (not included)

### Sustained behavior change shows higher rates of health improvement

**Fig. 2:**
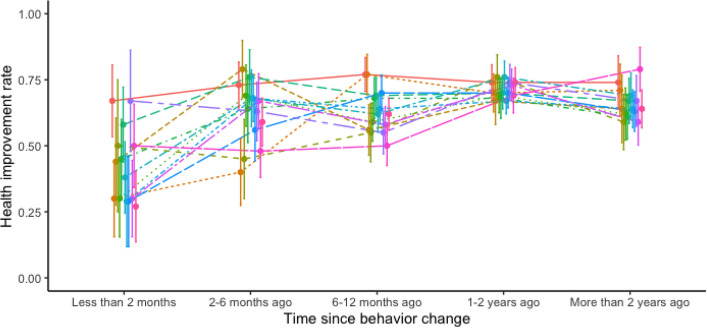
Health improvements as a function of behavior change duration. The graph depicts the rate of improvement related to the length of individual behavior changes (*n* = 859). Error bars represent standard error. Individual graphs for each behavior are shown in Figure S3

Time since the adoption of the behavior change was also captured in the surveys (Figure S2). Aggregated behavior changes with a duration longer than two months resulted in higher health improvement rates (2–6 months 0.63 ± 0.06, 6–12 months 0.63 ± 0.04, 1–2 years 0.71 ± 0.03, > 2 years 0.65 ± 0.02) than those below two months (0.43 ± 0.04) (*n* = 594, χ2_4_ = 41.95, *p* < 0.001) (Fig. 2 and Figure S3). A chi-square analysis excluding the lowest time point (< 2 months) still showed significance (*n* = 535, χ2₃ = 11.27, *p* = 0.01). Results exhibited higher variability in shorter time frames, both within each behavior and between them. However, variances narrowed at longer time frames, especially in periods longer than 12 months, and variability between different behaviors also decreased. At the time of the survey, all participants had received their reports for at least a year.

### Initial habits affect behavior change

**Fig. 3:**
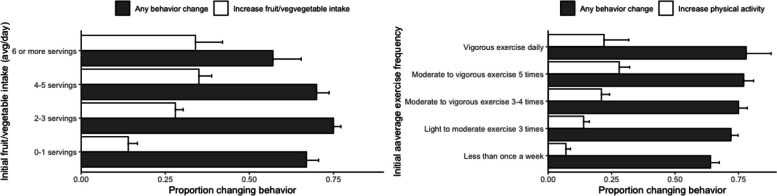
Proportion of participants adopting behavior changes stratified by baseline behaviors. Behavior changes stratified based on initial reported fruit and vegetable intake (**A**) and exercise frequency (**B**). Users who selected no need for any of the listed health improvements were removed from this visualization (*n* = 753). White bars indicate the proportion of related behavior changes (increases in fruit/vegetable intake (**A**) and physical activity (**B**)), and grey bars show the proportion of users selecting any of the behavior change options. Error bars represent standard error

From the initial questionnaire collected at onboarding (Figure S2), a three-factor ANOVA was performed to test the effects of exercise, fruit/vegetable intake, and restaurant frequency on baseline BMI, revealing significant associations with exercise (F(4, 847) = 29.74, *p* < 0.001), fruit/vegetable intake (F(3, 847) = 5.65, p < 0.001), and eating out frequency (F(5, 847) = 2.38, *p* = 0.037). No significant interaction was observed between exercise and fruit/vegetable intake (F(12, 847) = 0.60, *p* = 0.84). Behaviors at baseline also influenced behavior changes, as demonstrated by the follow-up survey. Specifically, intake of fruits and vegetables at baseline had a significant impact on behavior change related to an increase in those foods (*n* = 863, χ2₃ = 16.14, *p* = 0.001), as did initial exercise habits and an increase in physical activity (*n* = 863, χ2_4_ = 23.37, *p* < 0.001). Those with the most room for improvement changed the least in the relevant behavior. When examining fruit and vegetable intake, it was observed that users in the lowest category (0–1 servings per day) were the least likely to increase this behavior, with only 15% (CI ± 5.1%) of those participants reporting an increase in fruit and vegetable intake. This increase was almost half of what was observed in the other groups (Table S4 and Fig. 3A). Interestingly, general behavior changes also showed significant differences (*n* = 859, χ2_3_ = 9.02, *p* = 0.029), but within a narrower range (57%−73%) and in the opposite trend. As expected, there was a positive correlation between servings of fruits and vegetables reported at onboarding and the proportion of people who mentioned during the follow-up survey not needing any of the health improvement options given (*n* = 863, χ2_3_ = 16.81, *p* < 0.001). By removing this last group from the analysis, the proportion of people reporting increased fruit and vegetable intake showed a remarkable gradient correlating to the number of servings reported at onboarding (Fig. 3A). Similar results were observed for the frequency/intensity of physical activity (Table S4 and Fig. 3B). Unlike the analysis of fruit and vegetable intake, general behavior change did not show significant differences among exercise groups (*n* = 859, χ2_4_ = 5.03, *p* = 0.285). The specific behavior changes, however, had the same results, where those with the most room for improvement showed the lowest rates of related behavior change (Fig. 3B). In both groups, there were significant differences in sex (*n* = 862, χ2_3_ = 11.48, *p* = 0.009; *n* = 862, χ2_4_ = 21.44, p < 0.001), with higher female proportions reporting higher consumption of fruits and vegetables and males reporting higher levels of physical activity. In the case of fruit and vegetable intake, a significant age difference was also observed (F(3, 859) = 4.37, *p* = 0.004), with younger individuals consuming fewer servings of fruits and vegetables (Table S4).

## Discussion

This study aimed to assess the impact of behavior changes among nutrition-focused DTC-GTS users on health improvement. Self-reported health improvement rates were notably higher among those who made a behavior change. There was a dichotomy regarding weight change, with those who changed their behavior observing, on average, weight loss, compared to an average weight gain for those who did not change their behavior. This is consistent with the impact of some dietary behaviors on weight gain [24]. Furthermore, genotypes in key polymorphisms related to carbohydrate/fat intake (*FTO* rs9939609 A/, rs8050136 A/, *LEPR* rs1137101 G/) [6, 8, 9], showed a significant effect in weight loss after an increase in fruit and vegetable intake, which would likely correlate to an increase in carbohydrates (including fiber) intake and a decrease in total fat consumption, even though *PPARG* rs1801282 G/, another polymorphism related to obesity and fat intake [10], did not show significant change. Saturated fat intake was qualitatively inferred from a question regarding unhealthy snacking habits, which, in addition to saturated fat, also included added salt and sugars. Expected genotype effects were observed from G carriers in the *LEPR* polymorphism as well as in the effect alleles of the *FTO* polymorphisms, but not in other ones related to fat intake (*PPARG* rs1801282 G/, rs7903146 C/, *APOA2*, and rs5082 GG) [10–12]. In the case of rs5082, it is worth noting that the observation of decreased weight has been tested before[12], exclusively related to saturated fat. Still, in the present study, it was inferred from a question that would have also included refined carbohydrates. It will be needed to further dissect that trait with more nuance about different kinds of snacking. Interaction p-values were not included in the analysis, as all exceeded the threshold of statistical significance (p > 0.05). Importantly, the study was not powered or structured to detect novel interactions; rather, the objective was to evaluate whether providing participants with information about established gene–diet interactions could aid in making dietary choices and yield observable benefits. In addition, because behavior change was assessed in directional and qualitative terms, reporting interaction p-values would have limited interpretive value.

A prior large-scale study assessing dietary patterns among DTC-GTS users across the US found that a small proportion of the study participants met the recommended federal guidelines for different food groups [27], highlighting the need to develop strategies to improve dietary behaviors. Prior studies examining the effect of genetic testing on healthy behavior changes have yielded moderate results [3, 4, 28] and occasionally failed to demonstrate any differences [25]. Dietary behavior, for instance, was seen to change in 33% of users in one study [26]. The present study was not structured to assess the rates of behavior change accurately. An overestimation of behavior change rates is likely, as people who purchase a personalized nutrition genomics solution are likely to be already predetermined to make behavior changes. Furthermore, unlike most genetic testing services, the solution assessed in this study is designed to facilitate behavioral changes by providing dietary recommendations based on users' genetic profiles. This system aims to help users understand the rationale behind each recommendation, enabling them to decide which ones they want to follow. Focusing on actionable genetic traits accompanied by concrete recommendations has effectively facilitated sustainable behavior change [27]. The overwhelmingly positive impact on health outcomes that comes from the dietary behavior changes included in the study is likely to translate to broader populations, especially when those behavior changes are matched with key genotypes in well-understood variants. Adopting healthy behaviors, such as increased intake of fruits and vegetables, has been linked to positive health outcomes, including weight loss and a sense of well-being [28], which in turn reduces the probability of all-cause mortality [29].

Studies based on self-reported surveys rely on the respondents' perception and truthfulness in their responses, which can contain a degree of bias [30], especially when responses pertain to energy intake and weight [31]. The value and validity of self-report dietary data, however, have been thoughtfully acknowledged [32], highlighting important contributions that have emerged from these approaches, such as the correlation between red meat intake and increased mortality risk [33, 34]. This study showed strong associations between BMI and different behaviors at onboarding (fruit and vegetable intake, exercise frequency, and eating out frequency). Healthier behaviors were correlated with lower BMIs, which provides some validation for the truthfulness of the responses. Younger users tended to find the application more useful, consistent with the observation that younger customers also tend to comprehend genetic results more readily [14], a segment of the population less prone to self-report bias [35].

In some important behaviors (exercise and intake of fruits and vegetables), those with the most room for improvement changed the least in the relevant behavior. This reveals a peculiar pattern that may be related to the goal-gradient hypothesis, which proposes that more effort is invested when the goal is nearer [36]. Our results suggest that individuals already performing well in certain behaviors are more likely to continue progressing, which may already be yielding positive outcomes (the goal). However, among those with ample room for improvement, initial changes occur less frequently (when the goal might appear distant). This observation presents an attractive opportunity to focus additional effort on helping people overcome initial hurdles in adopting healthier behaviors, especially since rates for general behavior change have remained somewhat constant. This indicates that a general willingness to change behavior is present. A special focus on those hurdles could have a very impactful effect on improving well-being.

Generally, behavior changes showed stronger correlations to health improvements as they became sustained. This can be explained in two ways. It is likely that the longer a behavior change is carried out, the more likely a health benefit will result from it, as sustained behaviors are key [37]. At the same time, people are more likely to stick to a behavior change if, at early stages, they see their effort rewarded with a noticeable health improvement [38]. Finally, 29.8% of those users who did not change their behavior stated in a follow-up question that their primary reason for acquiring the service was to learn more about themselves, rather than to make changes (data not shown). This finding is consistent with a prior study that also showed customers value the explanatory nature of genetic tests [39].

Previous studies have shed light on the effectiveness of assistance through mobile and wireless technologies, yielding mixed but promising results [40]. It has been highlighted, however, that modest participation numbers (< 96) and short follow-up periods (< 6 months) in these instances underscore the need for larger studies with longer time frames [18]. Personalized nutrition DTC-GTS solutions easily accessible by mobile and web-based methods can serve as tools in aiding individuals to make behavior changes towards their health goals by providing information about their genetic-based nutritional needs in one location.

### Limitations

All study participants belonged to GenoPalate's user base; therefore, a control group without access to a DTC-GTS is lacking. Furthermore, it is very conceivable that people who purchase a genetic-based personalized nutrition service like GenoPalate, are already in a stage of change or motivated to make changes. Thus, behavior change rates would likely look different among the general population. GenoPalate’s user base is skewed towards white females. This demographic also dominated the study’s participants, which, together with the modest survey response rate (1.8%), represents a limitation of this study, as it may introduce response bias, increasing the representation of those users who remain engaged with the service and limiting the generalizability of the findings to the broader population. All data collected came from web-based self-report surveys, which can be less reliable than in-person assessments. The study focused on positive outcomes, and the results indicate that a segment of participants did not resonate with the genetic report. On average, lack of action led to weight gain, and it would be interesting to know if there were any negative outcomes beyond that.

## Conclusion

Behavioral changes among DTC-GTS users lead to health improvements consistent with expectations based on genetic results. This suggests that genetic testing services combined with personalized nutritional guidance can provide support for attainable and sustainable dietary and lifestyle changes, ultimately improving overall health and reducing the risk of chronic metabolic diseases.

## Supplementary Information

Supplementary Material 1

Supplementary Material 2

Supplementary Material 3

## Data Availability

The datasets generated during the current study are not publicly available due to GenoPalate’s privacy policy in which it is stated that their data is not used by third parties. Limited dataset can be available from the corresponding author on reasonable request.

## Abbreviations

ANOVA: Analysis of variance
BMI: Body mass index
CI: Confidence intervals
DTC-GTS: Direct-to-consumer genetic testing services
GENOFAM: Genetic-enabled nutritional output toward food as medicine
SD: Standard deviation
SNPs: Single nucleotide polymorphisms
