# Supplementary material for: Enhancing health outcomes through genetic-based personalized nutrition: investigating the effects of dietary behavior change
Source: BMC Nutr. 2026 Apr 7;12:96. doi: 10.1186/s40795-026-01311-6 (PMC13192085; doi:10.1186/s40795-026-01311-6)
Supplement: Supplementary file 1 — Supplementary Material 1 [file 40795_2026_1311_MOESM1_ESM.docx]

**
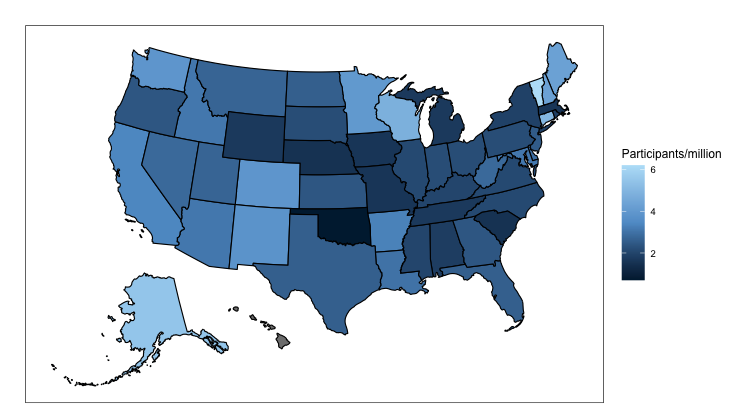
**

**Figure S1.** Survey respondents’ distribution across the USA adjusted by population. States in grey (HI) denote no participants.

**
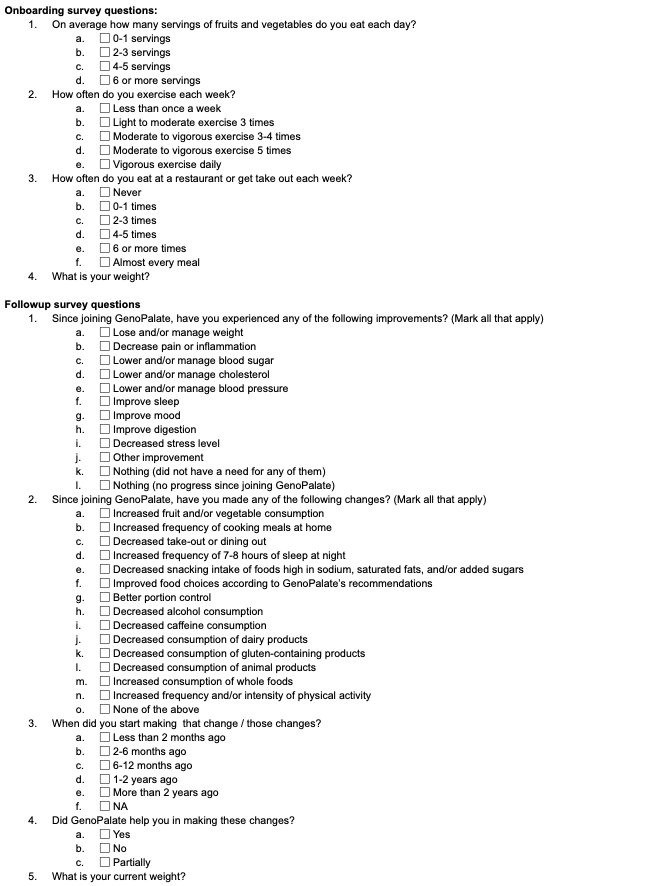
**

**Figure S2.** Onboarding and follow-up survey questions used in the study.


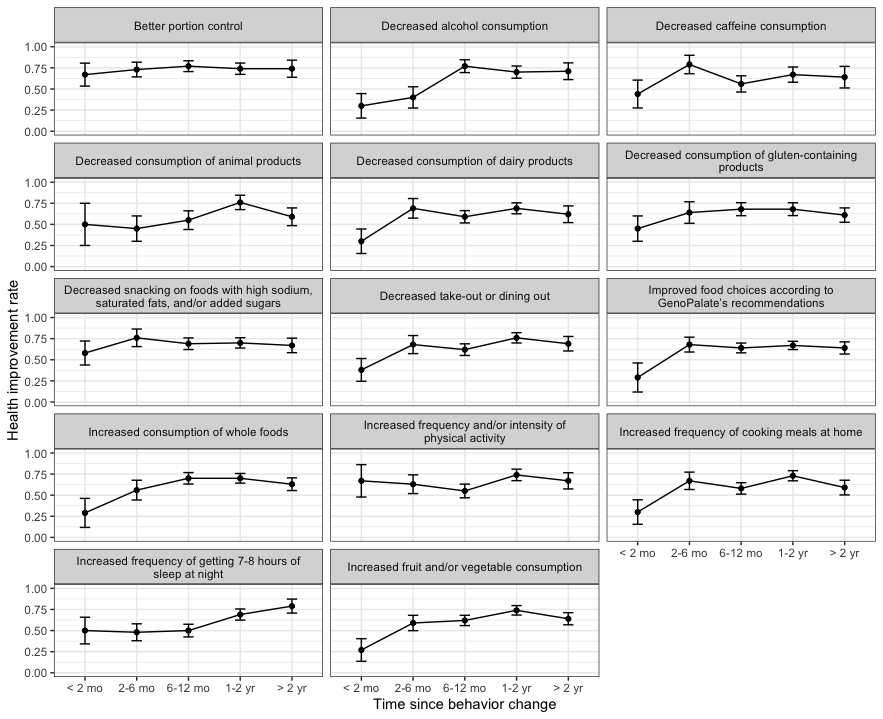


**Figure S3. Health improvement rate related to individual behavior changes.** The graph depicts the rate of

Reported health improvement related to the length of each behavior change (n = 859). Error bars represent SE.
