# Supplementary material for: Enhancing health outcomes through genetic-based personalized nutrition: investigating the effects of dietary behavior change
Source: BMC Nutr. 2026 Apr 7;12:96. doi: 10.1186/s40795-026-01311-6 (PMC13192085; doi:10.1186/s40795-026-01311-6)
Supplement: Supplementary file 2 — Supplementary Material 2 [file 40795_2026_1311_MOESM2_ESM.docx]

**Additional Tables**

**Table S1.** Impact of incentives on cohort characteristics

|  | No Incentive | Incentive | p |
| --- | --- | --- | --- |
| N (%) | 627 (72%) | 247 (28%) |  |
| Female, n (%) | 453 (72%) | 183 (74%) | 0.666* |
| Behavior change, p ± 95% CI | 0.67 ± 0.037 | 0.73 ± 0.055 | 0.137* |
| GenoPalate helpfulness, p ± 95% CI | 0.77 ± 0.033 | 0.83 ± 0.047 | 0.209* |
| Health improvement, p ± 95% CI | 0.41 ± 0.038 | 0.48 ± 0.062 | 0.08* |
| No need for health improvement, p ± 95% CI | 0.11 ± 0.024 | 0.15 ± 0.045 | 0.151* |
| BMI, mean ± sd | 29.1 ± 6.4 | 29.6 ± 6.9 | 0.38^‡^ |
| Age, mean ± sd | 54.2 ± 12.9 | 44.9 ± 12 | **<0.001^‡^** |

BMI measured in kg/m^2^. Significant differences are highlighted in bold. P denotes proportion. CI denotes confidence intervals with an alpha of 0.05.

^*^ Chi-square used to obtain p-values.

^‡^ Student t used to obtain p-values.

**Table S2.** Behavior change breakdown

|  | N (%) | Health improvement, p ± 95% CI | GP helped, p ± 95% CI | No need for health improvement, p ± 95% CI | Avg numb of improvements, p ± 95% CI | Female, n (%) | BMI, mean ± sd | Weight change (kg), mean ± sd | Weight change (%), mean ± sd | Age, mean ± sd |
| --- | --- | --- | --- | --- | --- | --- | --- | --- | --- | --- |
| Better portion control | 146 (17%) | 0.75 ± 0.07 | 0.83 ± 0.061 | 0.04 ± 0.032 | 2.2 ± 2.1 | 92 (63%) | 31.8 ± 7.3 | -2.5 ± 9.4 | -2.2 ± 9.5 | 51.3 ± 12.5 |
| Decreased alcohol consumption | 122 (14%) | 0.64 ± 0.085 | 0.74 ± 0.078 | 0.07 ± 0.045 | 1.9 ± 2.2 | 87 (71%) | 29.8 ± 5.8 | -1.8 ± 8.4 | -1.6 ± 9.5 | 51.6 ± 13.1 |
| Decreased caffeine consumption | 97 (11%) | 0.63 ± 0.096 | 0.78 ± 0.082 | 0.06 ± 0.047 | 1.7 ± 2.1 | 76 (78%) | 29.4 ± 7.1 | -0.6 ± 8.5 | 0 ± 9.3 | 51 ± 14.5 |
| Decreased consumption of animal products | 87 (10%) | 0.6 ± 0.103 | 0.76 ± 0.09 | 0.07 ± 0.054 | 1.8 ± 2.2 | 69 (79%) | 28.6 ± 6.6 | -1 ± 8.1 | -0.8 ± 8.9 | 55.1 ± 12 |
| Decreased consumption of dairy products | 151 (17%) | 0.62 ± 0.077 | 0.84 ± 0.058 | 0.05 ± 0.035 | 1.8 ± 2.1 | 113 (75%) | 29.4 ± 6.5 | -0.5 ± 7.4 | -0.2 ± 8.8 | 52.9 ± 11.7 |
| Decreased consumption of gluten-containing products | 138 (16%) | 0.63 ± 0.081 | 0.76 ± 0.071 | 0.04 ± 0.033 | 1.7 ± 2 | 110 (80%) | 29.2 ± 7.2 | -0.6 ± 8.7 | 0.3 ± 9.1 | 51.8 ± 13.4 |
| Decreased unhealthy snacking | 165 (19%) | 0.7 ± 0.07 | 0.85 ± 0.054 | 0.05 ± 0.033 | 2.1 ± 2.1 | 116 (70%) | 30.1 ± 6.9 | -1.9 ± 7.2 | -1.8 ± 8 | 53.3 ± 13.3 |
| Decreased take-out or dining out | 164 (19%) | 0.66 ± 0.073 | 0.78 ± 0.063 | 0.04 ± 0.03 | 1.9 ± 2.1 | 116 (71%) | 30.8 ± 7.5 | -2.6 ± 8.6 | -2.3 ± 9 | 53.1 ± 12.9 |
| Improved food choices according to  GenoPalate’s recommendations | 247 (28%) | 0.64 ± 0.06 | 0.96 ± 0.024 | 0.11 ± 0.039 | 1.7 ± 1.9 | 184 (74%) | 28.6 ± 7.1 | -0.8 ± 7.1 | -0.5 ± 8.1 | 51.1 ± 13.2 |
| Increased consumption of whole foods | 178 (20%) | 0.65 ± 0.07 | 0.84 ± 0.054 | 0.07 ± 0.037 | 1.8 ± 2 | 132 (74%) | 29.5 ± 6.8 | -1.1 ± 7.5 | -0.6 ± 8.3 | 53 ± 14.7 |
| Increased freq and/or intensity of physical activity | 130 (15%) | 0.65 ± 0.082 | 0.73 ± 0.076 | 0.07 ± 0.044 | 2 ± 2.2 | 89 (68%) | 29.6 ± 6.7 | -2.2 ± 8.4 | -2 ± 8.7 | 52.6 ± 11.9 |
| Increased freq of cooking meals at home | 176 (20%) | 0.62 ± 0.072 | 0.79 ± 0.06 | 0.07 ± 0.038 | 1.7 ± 1.9 | 125 (71%) | 30.6 ± 7.8 | -2.3 ± 9 | -1.9 ± 9.4 | 51.9 ± 15.1 |
| Increased freq of 7-8 hours of sleep at night | 158 (18%) | 0.61 ± 0.076 | 0.79 ± 0.064 | 0.06 ± 0.037 | 1.8 ± 2 | 113 (72%) | 30.1 ± 7 | 0 ± 7.6 | 0.7 ± 8.1 | 52.7 ± 12.3 |
| Increased fruit and/or vegetable consumption | 216 (25%) | 0.64 ± 0.064 | 0.84 ± 0.049 | 0.08 ± 0.036 | 1.8 ± 2 | 157 (73%) | 30.3 ± 7.2 | -1.1 ± 7.2 | -0.9 ± 8.4 | 53 ± 15.3 |
| None of the above | 265 (30%) | 0.12 ± 0.039 |  | 0.19 ± 0.047 | 0.2 ± 0.6 | 193 (73%) | 29.2 ± 6.1 | 1.1 ± 5.3 | 1.2 ± 6.2 | 51.5 ± 12.5 |
| p-value |  | **<0.001*** |  | **<0.001*** | **<0.001^§^** | 0.246* | 0.0021^§^ | **<0.001^§^** | **<0.001^§^** | 0.564^§^ |
| p-value excluding control |  | 0.574* | **<0.001*** | 0.385* | 0.763^§^ | 0.193* | 0.0033^§^ | 0.067^§^ | **0.042^§^** | 0.581^§^ |

BMI measured in kg/m^2^. Unhealthy snacking refers to high salt, saturated fats and/or sugars. Significant differences highlighted in bold. P denotes proportion. CI denotes confidence intervals with an alpha of 0.05.

^*^ Chi-square used to obtain p-values.

^§^ 1-way ANOVA used to obtain p-values.

**Table S3.** Cohort characteristics related to exercise and eating out frequency stratified by behavior change adoption

|  | Entire cohort | No behavior change | Behavior change | p |
| --- | --- | --- | --- | --- |
| Initial average exercise activity |  |  |  | 0.334 |
| Less than once a week | 219 (25.1%) | 79 (29.3%) | 139 (23.2%) |  |
| Light to moderate exercise 3 times | 295 (33.8%) | 91 (33.7%) | 204 (34.0%) |  |
| Moderate to vigorous exercise 3-4 times | 194 (22.2%) | 55 (20.4%) | 139 (23.2%) |  |
| Moderate to vigorous exercise 5 times | 141 (16.1%) | 37 (13.7%) | 101 (16.8%) |  |
| Vigorous exercise daily | 25 (2.9%) | 8 (3.0%) | 17 (2.8%) |  |
| Initial eating out frequency |  |  |  | 0.428 |
| Never | 83 (9.5%) | 24 (8.9%) | 59 (9.8%) |  |
| 0-1 times | 369 (42.2%) | 116 (43.0%) | 253 (42.2%) |  |
| 2-3 times | 305 (34.9%) | 89 (33.0%) | 214 (35.7%) |  |
| 4-5 times | 78 (8.9%) | 30 (11.1%) | 47 (7.8%) |  |
| 6 or more times | 28 (3.2%) | 6 (2.2%) | 21 (3.5%) |  |
| Almost every meal | 11 (1.3%) | 5 (1.9%) | 6 (1.0%) |  |
| Indian | 4 (0.4%) | 3 (1.0%) | 1 (0.1%) |  |

^*^ Chi-square used to obtain p-values.

**Table S4.** Behavior changes according to baseline responses in related behaviors

| Persona | N (%) | Specific behavior change,  p ± 95% CI | Specific health improvement, p ± 95% CI | General behavior change,  p ± 95% CI | General health improvement, p ± 95% CI | No need for improvement in health,  p ± 95% CI | GenoPalate helped,  p ± 95% CI | Avg number of improvements, p ± 95% CI | BMI,  mean ± sd | Age,  mean ± sd | Female,  n (%) |
| --- | --- | --- | --- | --- | --- | --- | --- | --- | --- | --- | --- |
| **Fruit and vegetable intake** | |  |  |  |  |  |  |  |  |  |  |
| 0-1 servings | 187 (22%) | 0.14 ± 0.05 | 0.56 ± 0.071 | 0.66 ± 0.068 | 0.42 ± 0.071 | 0.1 ± 0.043 | 0.8 ± 0.057 | 0.9 ± 1.5 | 31.1 ± 6.3 | 49 ± 13.4 | 119 (64%) |
| 2-3 servings | 439 (51%) | 0.27 ± 0.042 | 0.62 ± 0.045 | 0.73 ± 0.042 | 0.44 ± 0.046 | 0.1 ± 0.028 | 0.79 ± 0.038 | 1 ± 1.5 | 29.6 ± 6.7 | 51.6 ± 13.9 | 323 (74%) |
| 4-5 servings | 190 (22%) | 0.32 ± 0.066 | 0.73 ± 0.063 | 0.66 ± 0.067 | 0.44 ± 0.071 | 0.18 ± 0.055 | 0.79 ± 0.058 | 1.1 ± 1.6 | 27 ± 5.5 | 53.4 ± 12.4 | 149 (78%) |
| 6 or more servings | 47 (5%) | 0.26 ± 0.125 | 0.5 ± 0.143 | 0.55 ± 0.142 | 0.3 ± 0.131 | 0.26 ± 0.125 | 0.81 ± 0.112 | 0.8 ± 1.7 | 27.9 ± 6.4 | 54.7 ± 10.2 | 36 (77%) |
| **p-value** |  | **0.001*** | 0.236* | **0.029*** | 0.319* | **p<0.001*** | 0.942* | 0.467^‡^ | **p<0.001^‡^** | **0.005^‡^** | **0.009*** |
|  |  |  |  |  |  |  |  |  |  |  |  |
| **Exercise regimen** |  |  |  |  |  |  |  |  |  |  |  |
| Less than once a week | 216 (25%) | 0.07 ± 0.034 | 0.69 ± 0.062 | 0.64 ± 0.064 | 0.39 ± 0.065 | 0.1 ± 0.04 | 0.78 ± 0.055 | 0.8 ± 1.4 | 31.9 ± 7 | 50.8 ± 13.1 | 175 (81%) |
| Light to moderate exercise 3 times | 292 (34%) | 0.13 ± 0.039 | 0.66 ± 0.054 | 0.69 ± 0.053 | 0.4 ± 0.056 | 0.11 ± 0.036 | 0.79 ± 0.047 | 1 ± 1.6 | 29.9 ± 6.4 | 52.3 ± 13.1 | 220 (75%) |
| Moderate to vigorous exercise 3-4 times | 189 (22%) | 0.2 ± 0.057 | 0.65 ± 0.068 | 0.72 ± 0.064 | 0.46 ± 0.071 | 0.13 ± 0.048 | 0.8 ± 0.057 | 1 ± 1.4 | 27.8 ± 5.7 | 50.3 ± 14.6 | 124 (66%) |
| Moderate to vigorous exercise 5 times | 141 (16%) | 0.24 ± 0.07 | 0.68 ± 0.077 | 0.73 ± 0.073 | 0.49 ± 0.083 | 0.14 ± 0.057 | 0.77 ± 0.069 | 1.2 ± 1.7 | 26.5 ± 5 | 53.1 ± 12.5 | 95 (67%) |
| Vigorous exercise  daily | 25 (3%) | 0.2 ± 0.157 | 0.4 ± 0.192 | 0.68 ± 0.183 | 0.4 ± 0.192 | 0.28 ± 0.176 | 0.88 ± 0.127 | 1.1 ± 2 | 24.9 ± 4.1 | 52.8 ± 12.7 | 13 (52%) |
| **p-value** |  | **p<0.001*** | 0.811* | 0.284* | 0.279* | 0.115* | 0.745* | 0.268^‡^ | **p<0.001^‡^** | 0.262^‡^ | **p<0.001*** |

BMI measured in kg/m^2^. Significant differences highlighted in bold. P denotes proportion. CI denotes confidence intervals with an alpha of 0.05.

^*^ Chi-square used to obtain p-values.

^‡^ 1-way ANOVA used to obtain p-values.
