# Supplementary material for: Enhancing health outcomes through genetic-based personalized nutrition: investigating the effects of dietary behavior change
Source: BMC Nutr. 2026 Apr 7;12:96. doi: 10.1186/s40795-026-01311-6 (PMC13192085; doi:10.1186/s40795-026-01311-6)
Supplement: Supplementary file 3 — Supplementary Material 3 [file 40795_2026_1311_MOESM3_ESM.pdf]

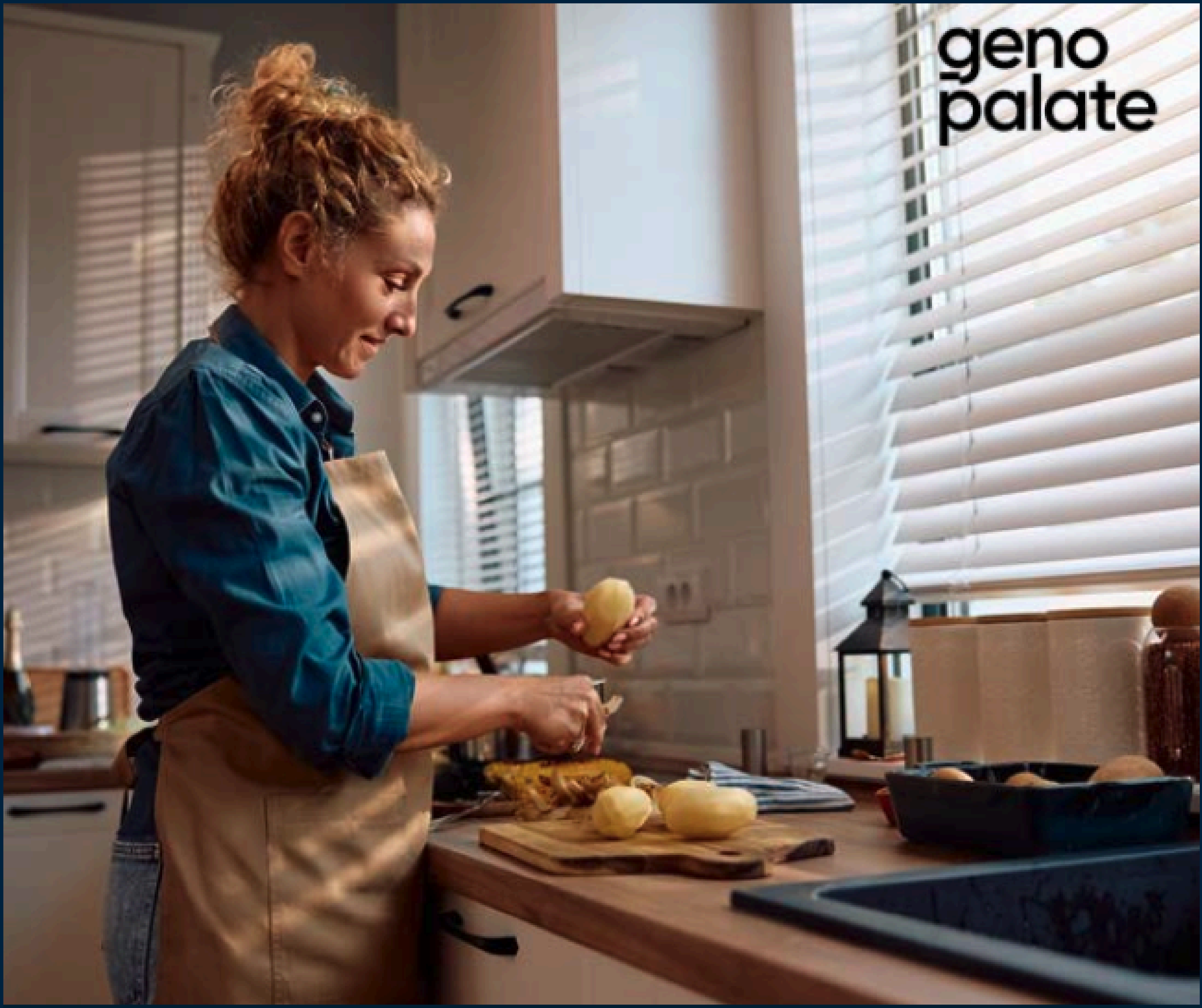

SAMPLE REPORT

Essential Nutrition Report

See how your unique genetic makeup shapes your ideal diet. Get a sneak peek into the insights our Essential Nutrition Report provides to help you eat smarter and feel your best.

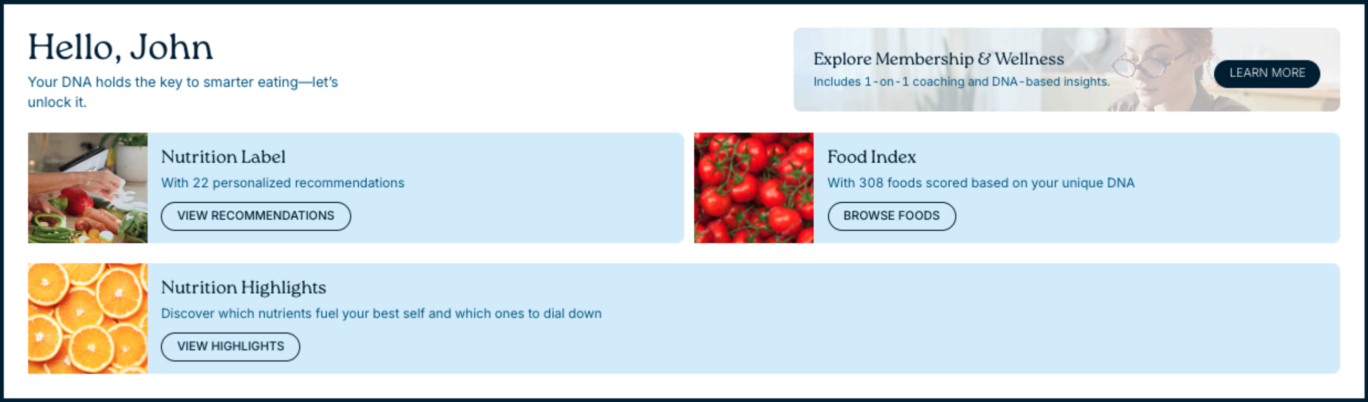

Home dashboard from where you can select which section to explore and find relevant content personalized to your health concerns and objectives.

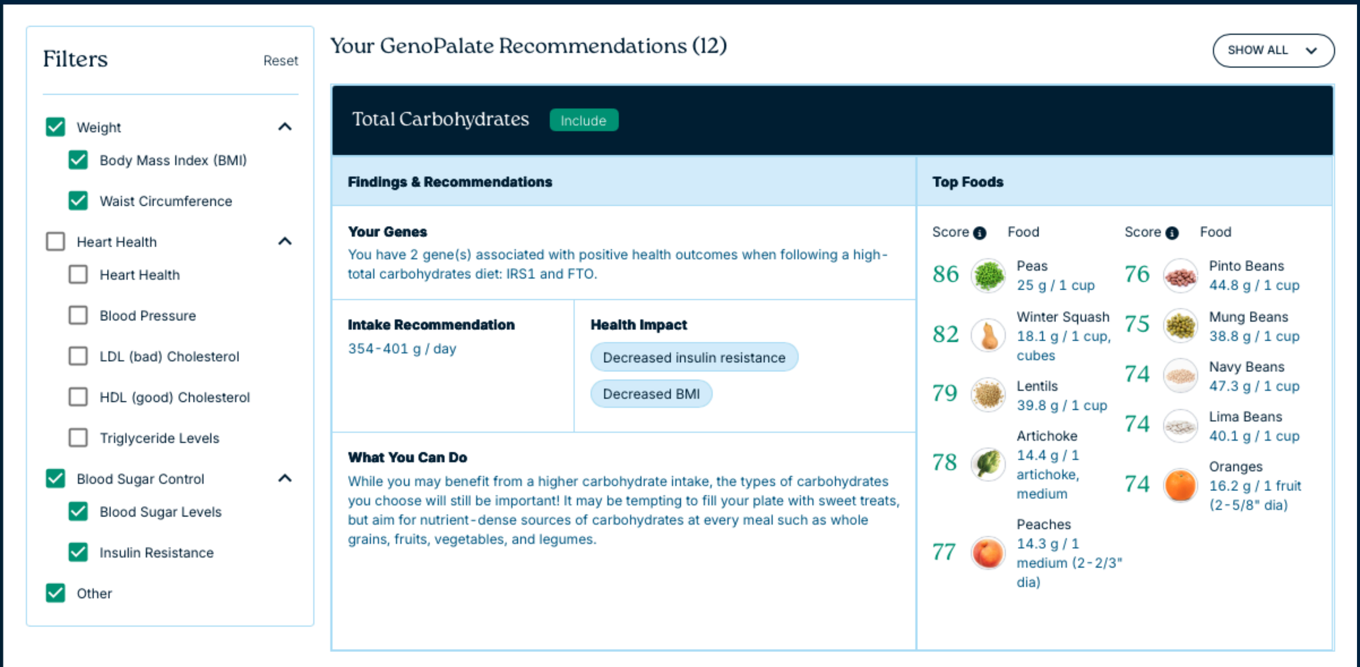

Highlights page with dynamic filtering, allowing you to find nutritional traits more relevant to you. Easily see what health impacts will have on you, based on your genetics, and immediately find food sources for that nutrient.

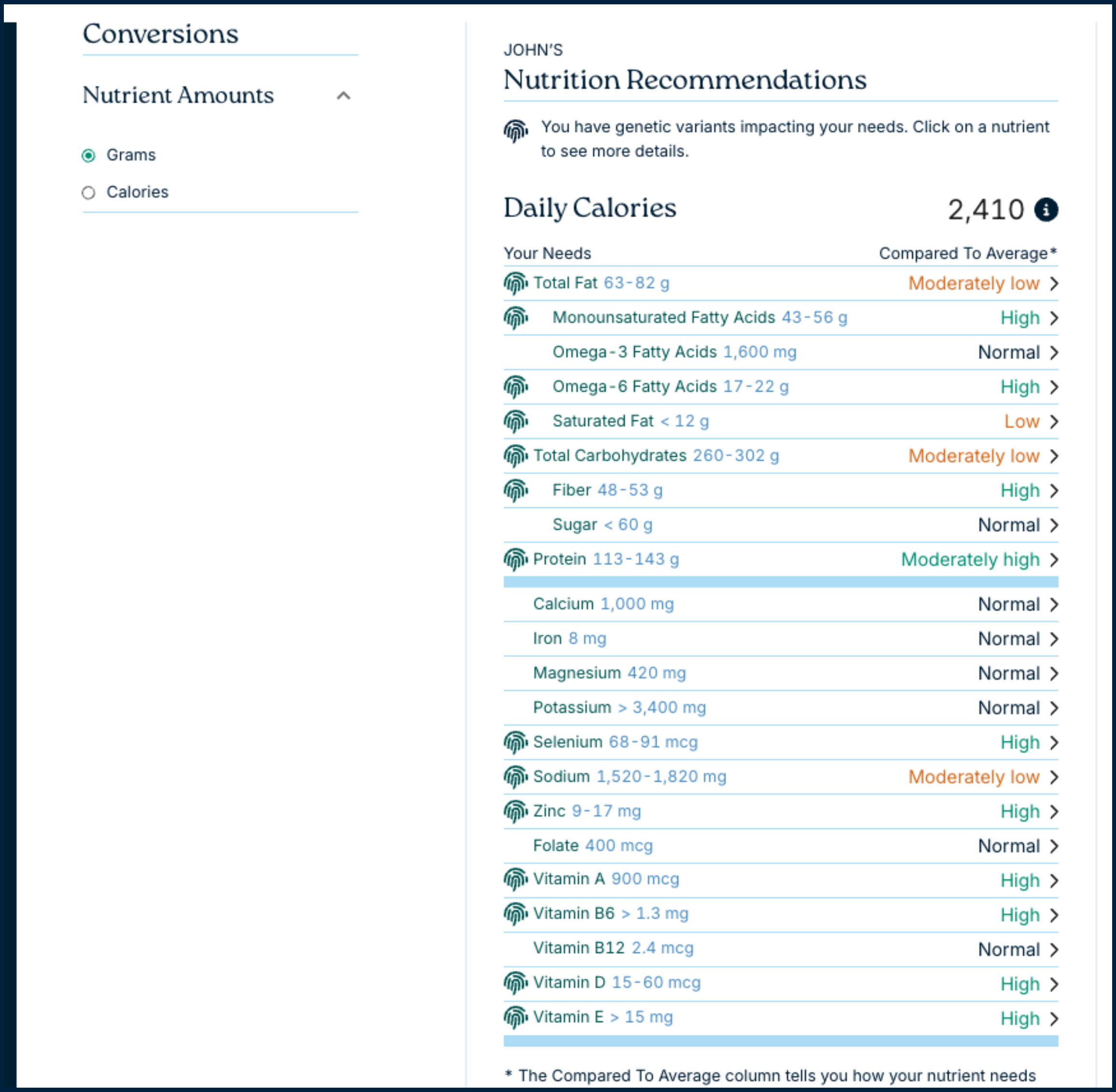

Personalized nutrition label to see your full nutrient needs. Easily find the amount you need from each nutrient and determine if you require less or more than the typical guidelines. Each nutrient is clickable, allowing you to explore it in more detail.

Fiber

John, individuals with your genes may benefit from getting at least 48-53 g of fiber per day.

Evidence supports that individuals who have your specific genes have the following benefits: improved blood sugar levels, decreased bmi when consuming at least 48-53 g of dietary fiber daily. In addition to these health benefits, aiming to consume the recommended DRI of 30 to 38 grams of fiber each day is associated with additional benefits including improved cardiovascular health, gut health, and decreased hunger.

While fiber falls within the carbohydrate category, it differs from other sources of carbohydrate because it cannot be broken down by the body. Also known as "roughage", fiber can be found in plant sources. For reference, one cup of mango has about 2.6 grams of fiber, one serving of carrots has 3.6 grams of fiber, and ½ cup of black beans has 3.7 grams of fiber.

Your recommended total daily caloric intake is 2410. Total daily calories are determined using your age, gender, height, weight, activity level, and health goals.

Overview | Genetic Impact

You have two of the genetic variants we tested for.

VARIANTS DETECTED | ALL TESTED MARKERS

MARKER	GENOTYPE	WHAT YOUR GENOTYPE MEANS	GENE INFORMATION	RESEARCH
Gene FTO<br>Marker GP000008	AC	We detected a variant that is linked to lower body mass index (BMI) when consuming a high-fiber intake.<br>40% of the population shares your genotype	The FTO gene affects your food cravings and how your body uses or stores food. Variants within this gene are linked to different body compositions.	References[2]
Gene TCF7L2<br>Marker GP000005	CC	We detected a variant that is linked to improved blood sugar levels when eating a high-fiber intake.<br>61% of the population shares your genotype	The TCF7L2 gene provides instructions for making a protein that helps balance your blood sugar.	References[1]

References

1. Hindy, G., Mollet, I. G., Rukh, G., Ericson, U., & Orho-Melander, M. (2016). Several type 2 diabetes-associated variants in genes annotated to WNT signaling interact with dietary fiber in relation to incidence of type 2 diabetes. Genes & Nutrition, 11(1). https://doi.org/10.1186/s12263-016-0524-8
2. Hosseini-Esfahani, F., Koochakpoor, G., Daneshpour, M. S., Mirmiran, P., Sedaghati-khayat, B., & Azizi, F. (2017). The interaction of fat mass and obesity associated gene polymorphisms and dietary fiber intake in relation to obesity phenotypes. Scientific Reports, 7(1), 1–9. https://doi.org/10.1038/s41598-017-18386-12

Discover which of your genes are driving the recommendation for a particular nutrient and explore the scientific literature behind it.

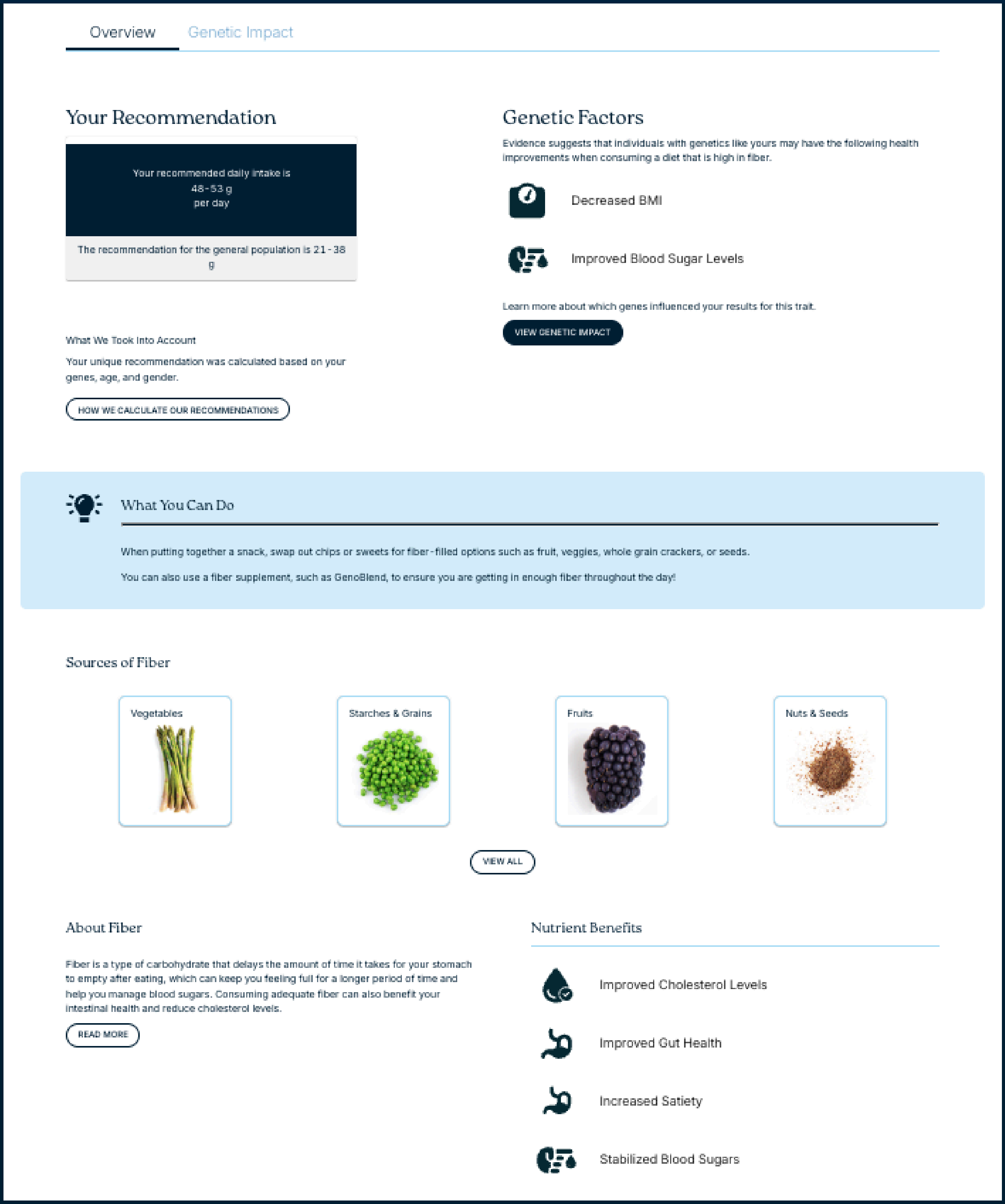

Learn more about the nutrient, including what you can do, its health benefits, and explore your food options to find the best sources.

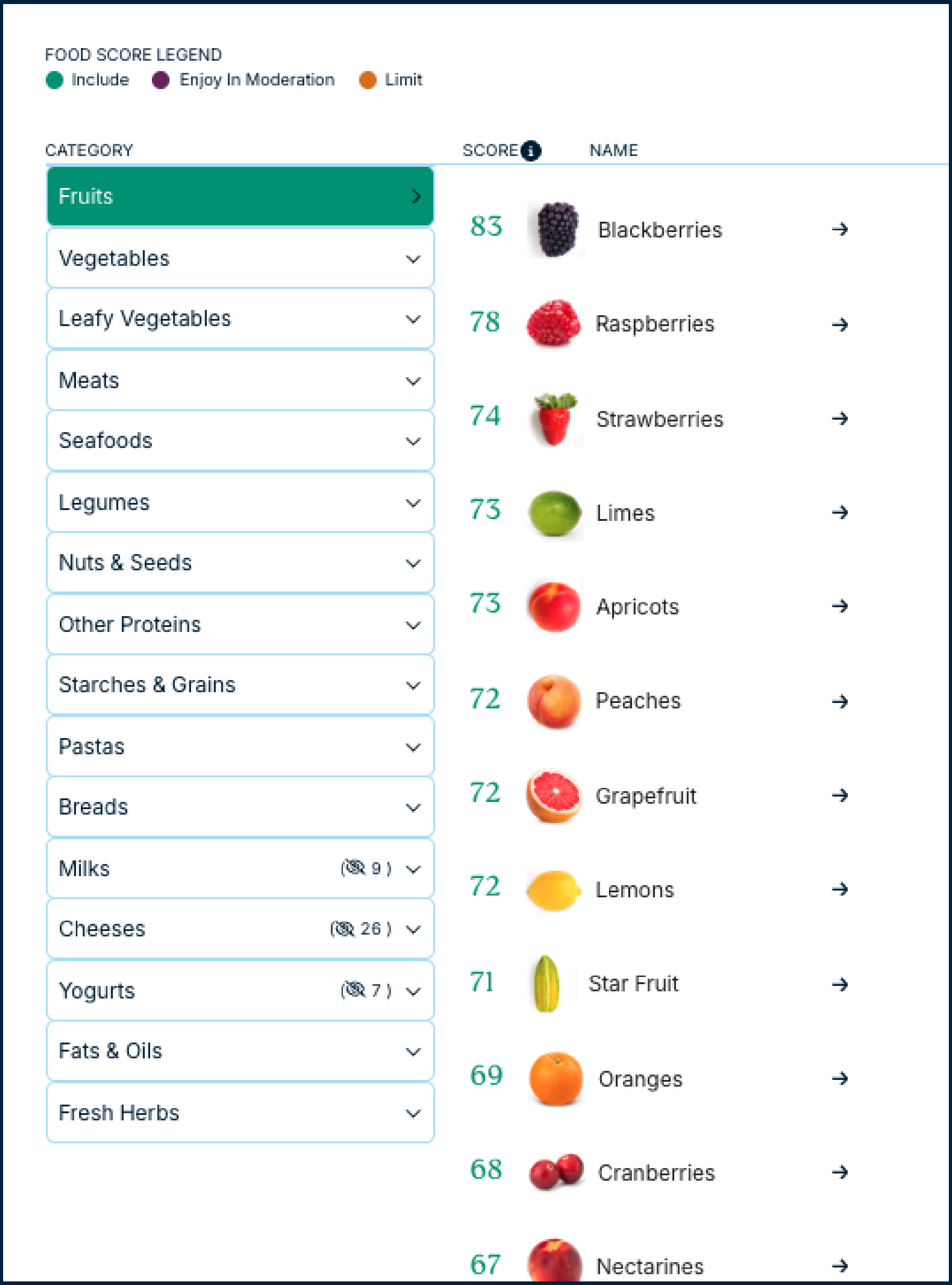

Discover which foods are your best options. They all contain a personalized score based on nutrient content and your genetic needs.
